# Fixation technique of biodegradable magnesium alloy suture anchor in the rotator cuff repair of the shoulder in a goat model: a technical note

**DOI:** 10.1186/s12891-024-07300-9

**Published:** 2024-03-28

**Authors:** Wei-Chien Hsu, Guan-Lin Wu, Ming-Long Yeh

**Affiliations:** 1 Department of Orthopedics, An Nan Hospital, China Medical University, Tainan, Taiwan; 2 Department of Biomedical Engineering, College of Engineering, National Cheng Kung University, Tainan, Taiwan; 3 Medical Device Innovation Center, National Cheng Kung University, Tainan, Taiwan

**Keywords:** Rotator cuff tear, Magnesium alloy, Suture anchor, Goat, Biodegradable

## Abstract

**Background:**

Shoulder disorders, particularly rotator cuff tears, are prevalent musculoskeletal conditions related to aging. Although the widely used suture anchor technique provides strong mechanical support to the tendon, it is associated with a risk of postoperative tendon retearing. The conventionally used titanium alloys can affect the interpretation of magnetic resonance imaging. Degradable magnesium alloys possess excellent biocompatibility, similar mechanical property to the bone, and stimulating bone formation ability from Mg^2+^. The purpose of this experiment was to develop innovative magnesium-based suture anchors to enhance rotator cuff repair by improving fixation materials, and to evaluate their feasibility in a goat model.

**Methods:**

We developed fluoridized ZK60 suture anchors as the implantation material for two goats, who underwent rotator cuff repair surgery on both shoulders. Computed tomography (CT) and histological analysis were performed at 12 weeks postoperatively, and the results were compared between the magnesium and titanium alloy groups. Additionally, a hematological examination was conducted, which included assessments of red blood cells, white blood cells, platelets, coagulation function, liver function, kidney function, and magnesium ion concentration.

**Results:**

The 12-week postoperative CT images showed intact MgF_2_ ZK60 suture anchors, effectively reconnecting the infraspinatus tendon to the humeral head. The anchors became less visible on CT scans, indicating absorption by surrounding tissues. New bone formation in the MgF_2_ group surpassed that in the Ti group, demonstrating superior osseointegration. The similarity between cortical bone and magnesium reduced stress-shielding and promoted bone regeneration. Histological analysis revealed successful tendon healing with MgF_2_ anchors, while the Ti group showed discontinuous interfaces and reduced collagen secretion. Hematological examination showed stable liver, renal function, and magnesium ion levels.

**Conclusions:**

The findings indicate that MgF_2_-coated suture anchors are feasible for rotator cuff repair and potentially other orthopedic applications. We hope that magnesium alloy anchors can become the solution for rotator cuff tendon repair surgery.

**Supplementary Information:**

The online version contains supplementary material available at 10.1186/s12891-024-07300-9.

## Background

Shoulder disorders are prevalent musculoskeletal conditions among aging individuals; in particular, rotator cuff tears affect a large patient population [1]. These tears often result in shoulder weakness, functional loss, and persistent pain. Their most common surgical treatment is the suture anchor technique [1]. This technique involves the placement of anchors, accompanied by sutures, into the greater tuberosity of the humerus. The sutures are then passed through the torn tendon, enabling its reattachment to the original site on the bone. This process enhances the contact area between the tendon and bone, thereby promoting rotator cuff regeneration and healing [2, 3]. This technique provides robust support to the tendon [4]. However, it is associated with a major risk of postoperative tendon retearing.

Titanium alloys have been conventionally used as suture anchors. In recent years, magnetic resonance imaging (MRI) has been commonly employed for the postoperative evaluation of rotator cuff repair. Because the anchors are not removed after surgery and titanium alloys do not degrade in the human body, they can interfere with the interpretation of MRI. Consequently, polymers have been gradually replacing titanium alloys. However, their mechanical strength is inferior to that of metals, and their degradation process can create an acidic microenvironment, leading to concerns such as tissue inflammation that can hamper healing [5].

Magnesium alloys exhibit outstanding biocompatibility; they are light and have high mechanical strength, properties that closely resemble those of bone. Moreover, their degradation products can stimulate bone formation [6–13]. They have therefore been attracting increasing recognition as the next generation of biodegradable metallic implants in orthopedics [6–11]. In this study, we employed a specially developed suture anchor composed of a magnesium, zinc, and zirconium (Mg-6.0Zn-0.5Zr, ZK60) alloy [5, 6, 9]. To enhance its corrosion resistance, a protective magnesium fluoride (MgF_2_) coating was deposited on the alloy surface [5, 6, 9]. We conducted relevant in vivo tests, which revealed that magnesium alloy anchors have promising biocompatibility for use in clinical practice. However, few study has evaluated the application of magnesium alloy anchors in rotator cuff tears by using suitable goat models [12]. Therefore, our purpose is to develop innovative magnesium-based suture anchors to enhance rotator cuff repair by improving fixation materials and evaluated its feasibility in a goat model. We also evaluated the metallic interference of these anchors on MRI scans. Our hypothesis is that magnesium alloy suture anchors are a safe and effective implant in the context of rotator cuff tendon repair surgery, promoting tendon healing.

## Methods

### Magnesium suture anchor

We used the ZK60 magnesium alloy (Johnson & Annie, New Taipei City, Taiwan) for fabricating the suture anchors. In the manufacturing process, CNC machining (HUANG LIANG Biomedical Technology, Taiwan) was employed to create anchors with a diameter of 5 mm and a length of 15 mm. Prior to use, the anchors were ultrasonically cleaned in ethanol and deionized water for 5 min. These untreated anchors are referred to as Mg in subsequent discussions.

### MgF_2_ coating treatment

**Fig. 1:**
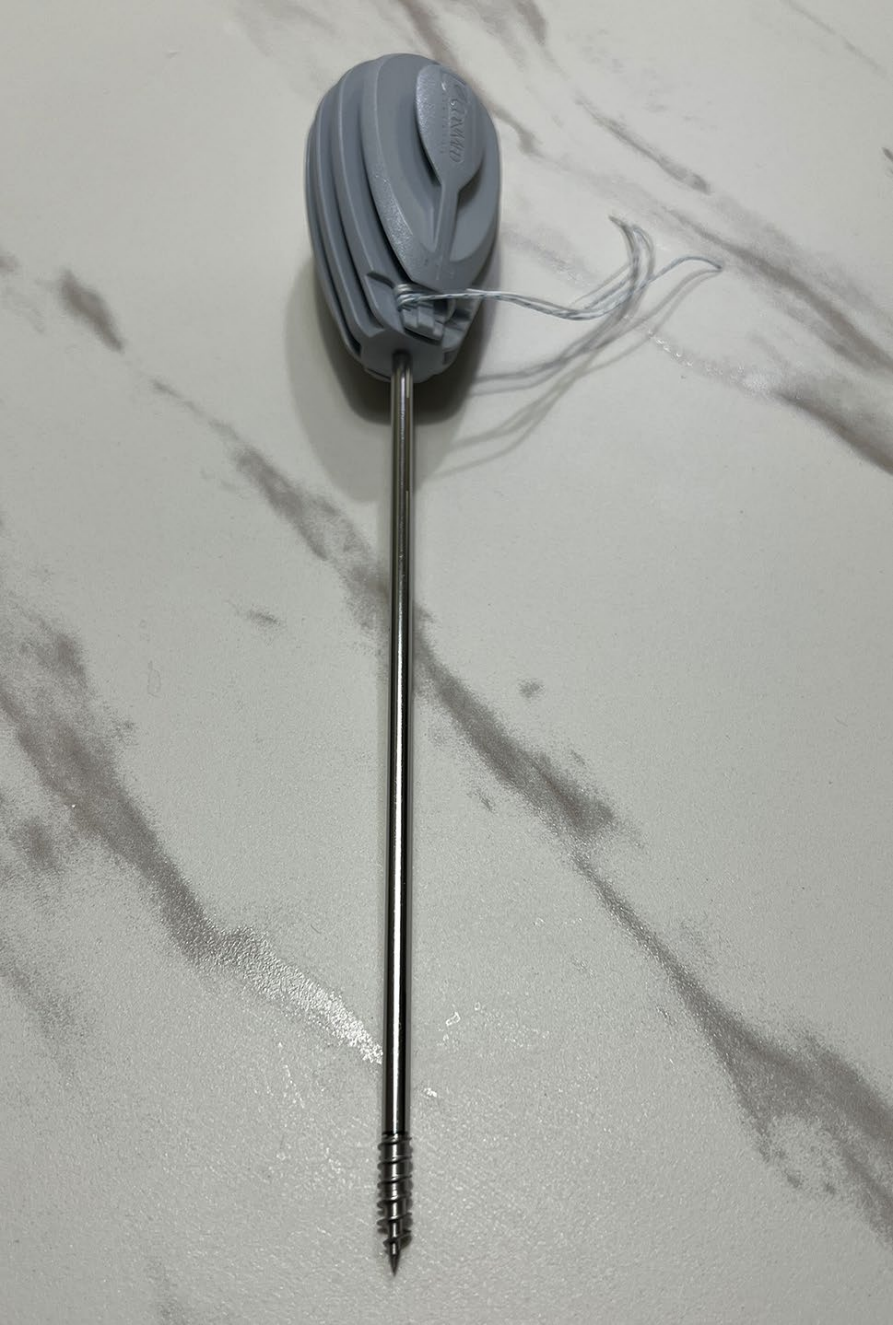
The photo of the MgF_2_ ZK60 suture anchor

To enhance the performance and corrosion resistance of the suture anchors, the fabricated ZK60 anchors were subjected to a fluoride coating treatment, in which the suture anchors were immersed in 42% hydrofluoric acid at room temperature with shaking at 70 rpm for 24 h on an orbital shaker [12–15]. This process resulted in the deposition of a nanoscale MgF_2_ coating layer on the alloy surface. Next, the anchors were again ultrasonically cleaned in ethanol and deionized water for 5 min. The coated anchors are denoted as MgF_2_ ZK60 suture anchors in subsequent discussions (Fig. 1).

### In vivo test

Two strands of #2 (5 metrics) suture wires were passed through the eyelet of each anchor. The control group consisted of commercially available titanium anchors (15 × 5 mm, Super Revo FT, CONMED, Largo, FL, USA), labeled as Ti in subsequent discussions. Before surgery, the MgF_2_ anchors, Ti anchors, and suture wires were sterilized with 75% ethanol.

### Animal model

The study protocol was approved by the Ethics Committee on Animal Use (IACUC approval No. NLAC(TN)-111-M-006) of National Laboratory Animal Center, National Applied Research Laboratories, Tainan, Taiwan. We selected two castrated male Boer goats: one weighed 38.84 kg and was 8 months old, and the other weighed 49.14 kg and was 1 year 2 months old. The goats were fed a mixture of alfalfa and oat hay (for approximately 3% of the body weight) to meet their nutritional needs. Additionally, Labdiet HF 5326 was supplied to goats with lower body condition scores, as determined by a veterinarian. Tap water was provided ad libitum throughout the study. During the research period, the goats were kept in an environment with 16–27 ℃, 30–70% humidity and a 12-h light/dark cycle.

### Surgery

Preoperative anesthesia medications included the intravenous administration of ketamine (4–5 mg/kg) and diazepam (0.3–0.5 mg/kg), subcutaneous administration of buprenorphine (0.01 mg/kg) and meloxicam (0.5 mg/kg), and intramuscular administration of metoclopramide (0.2–0.5 mg/kg) and penicillin G (10,000–60,000 IU/kg).

**Fig. 2:**
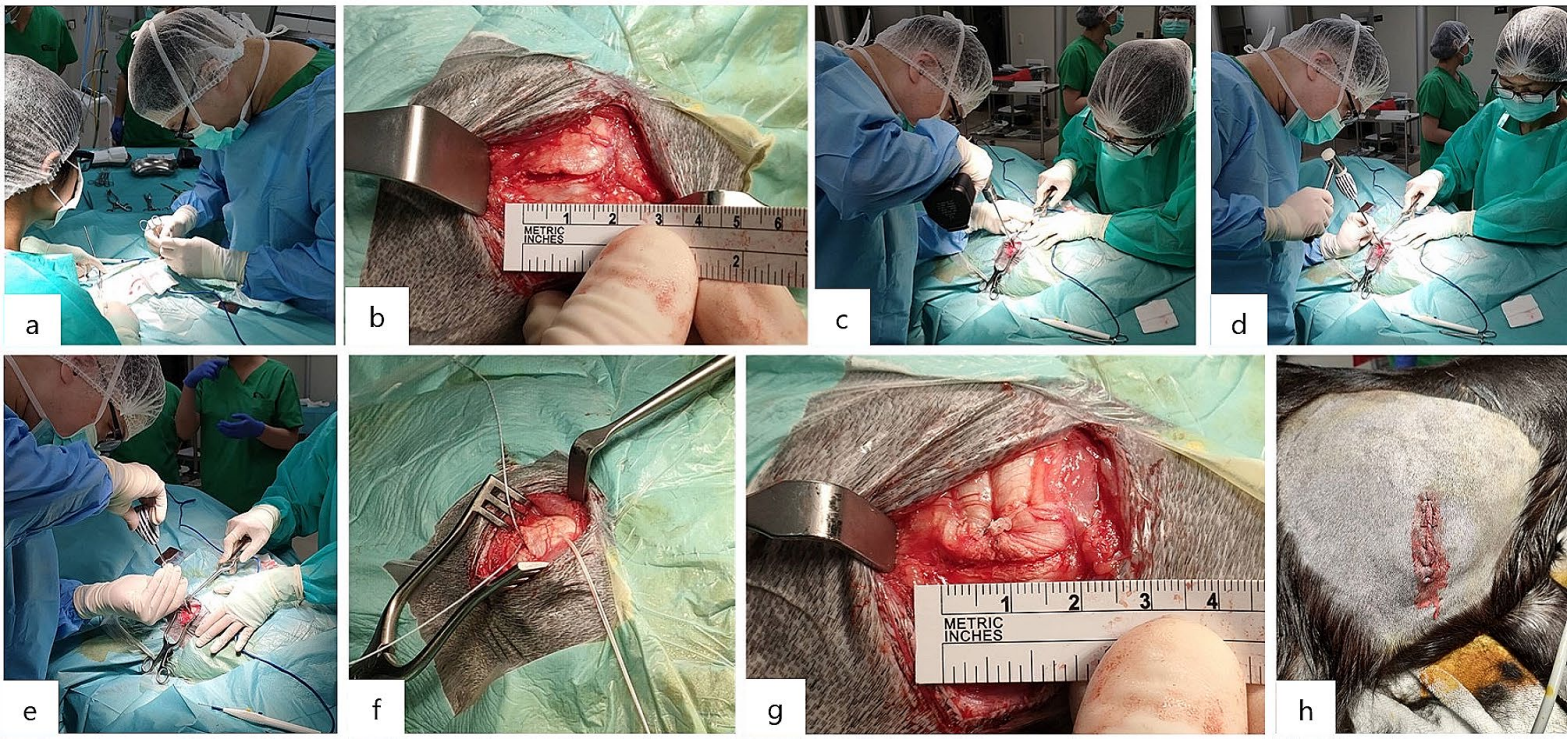
The Photos during surgery. (**a**) Blunt dissection was performed through the deltoid muscle to visualize the infraspinatus tendon. (**b**) We created a 2 cm iatrogenic full-thickness rotator cuff tear in the infraspinatus tendon. (**c**, **d**, **e**) After removing the articular cartilage and decorticating, a suture anchor was inserted at the junction area of the humeral head’s tuberosity. (**f**) The sutures passed through the anchor’s eyelet were attached to the torn tendon. (**g**) The detached cuff was repaired using the Mason–Allen method. (**h**) The incision was closed subcutaneously with 2 − 0 Vicryl and superficially with 3 − 0 Nylon

Prior to surgery, the shoulder areas of each goat were shaved and disinfected to ensure the proper aseptic surgical technique. A 5-cm incision was made parallel to the scapular spine and the infraspinatus tendon. Blunt dissection was performed through the deltoid muscle to visualize the infraspinatus tendon. The infraspinatus tendon was released using a No. 15 blade at the base of the tendon insertion area. We made a 2-cm iatrogenic full-thickness rotator cuff tear of the infraspinatus tendon. Following articular cartilage removal and decortication, a suture anchor was inserted into the junction area of the tuberosity of the humeral head [16–18]. The sutures passing through the eyelet of the anchor were attached to the torn tendon, and the detached cuff was repaired using the Mason–Allen method [19–21] (Fig. 2).

The intraoperative medications used were isoflurane inhalation (0.2–5%) and intravenous lactated Ringer’s solution (1–10 mL/kg/h). The postoperative care regimen included daily subcutaneous administration of enrofloxacin (5 mg/kg) and meloxicam (0.5 mg/kg) for 7 days.

**Fig. 3:**
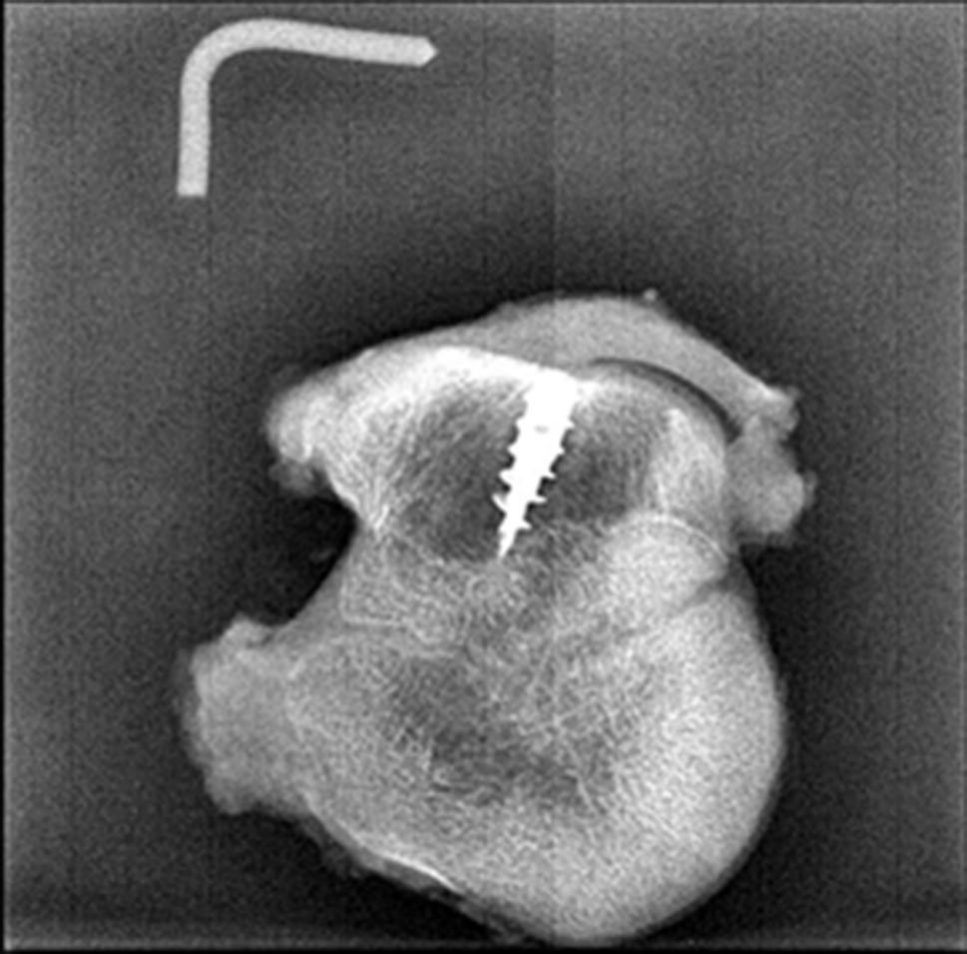
Postoperative X-ray revealing adequate positioning of the suture anchor and no displacement

**Fig. 4:**
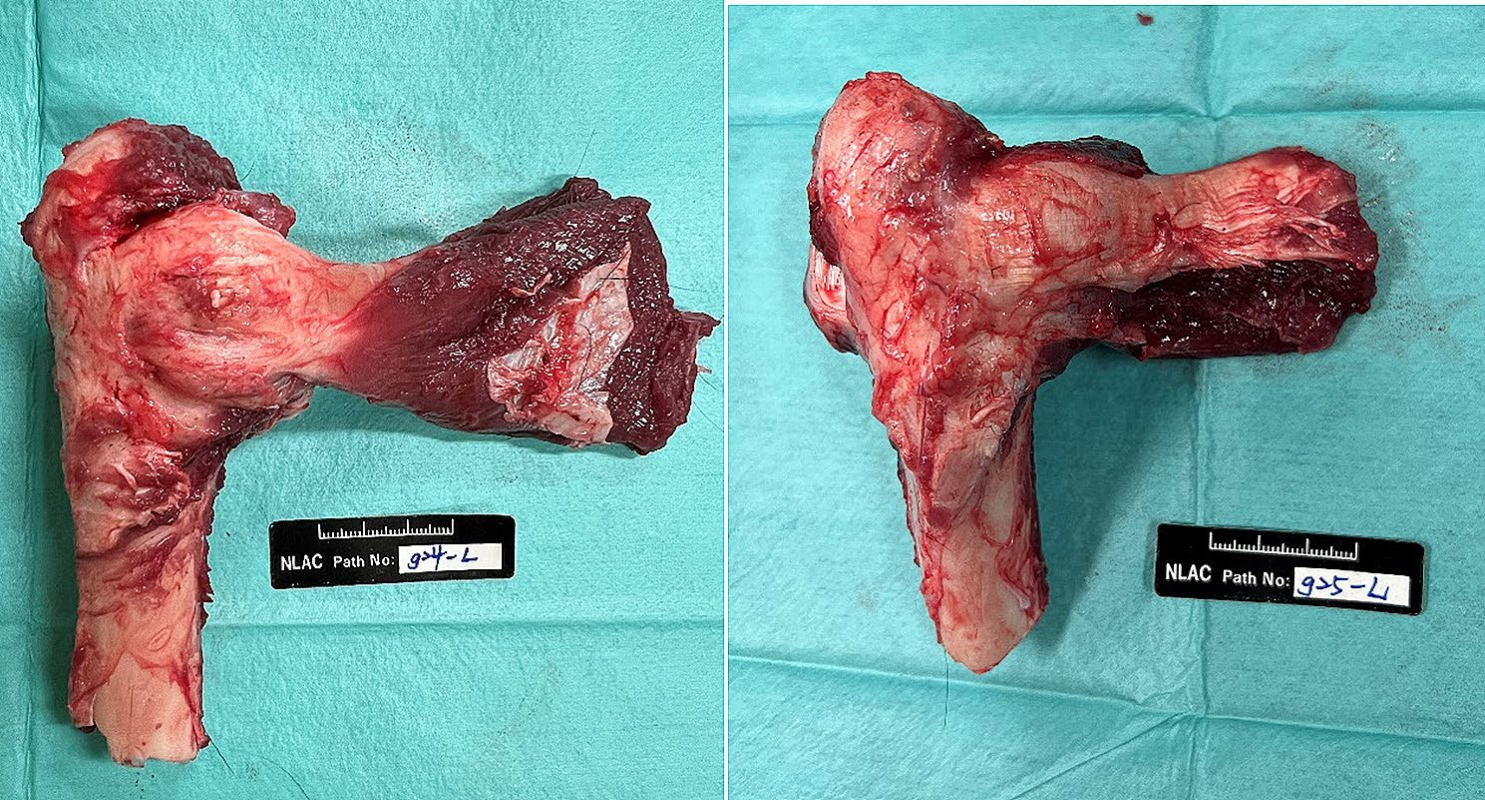
The specimen three months after surgery

After the surgical procedure, the position of the suture anchors was confirmed with X-rays (Fig. 3). Computed tomography (CT) scans and hematological examinations were conducted immediately, 4 weeks, and 12 weeks after surgery. At 12 weeks postoperatively, goats were euthanized for histological analysis. The soft tissue surrounding the bone was removed, except for the infraspinatus tendon (Fig. 4). Each extracted limb was preserved in 4% formaldehyde until further analysis. Following macroscopic examination, the sections were affixed to an acrylic sheet and ground to a thickness of approximately 50 μm. Subsequently, the samples were subjected to hematoxylin–eosin (H&E) staining. Histological analyses were then conducted using a motorized microscope system to observe bone–tendon interface healing, bone–implant interaction, and new bone growth.

The histology evaluation format of cell response for implant material scoring around the implant followed the guidelines outlined in Supplementary Table 1 of ISO 10993-6:2016. Similarly, the histology evaluation format of tissue response for implant material scoring around the implant adhered to the criteria specified in Supplementary Table 2 of ISO 10993-6:2016. An overall rating of the test samples was determined by combining the scores obtained from both evaluations, with a rating range of 0–15, as stipulated by the requirements of ISO 10993-6:2016. The rating categories included minimal or no reaction (0.0–2.9), slight reaction (3.0–8.9), moderate reaction (9.0–15.0), and severe reaction (> 15).

## Results

### Radiographic results

**Fig. 5:**
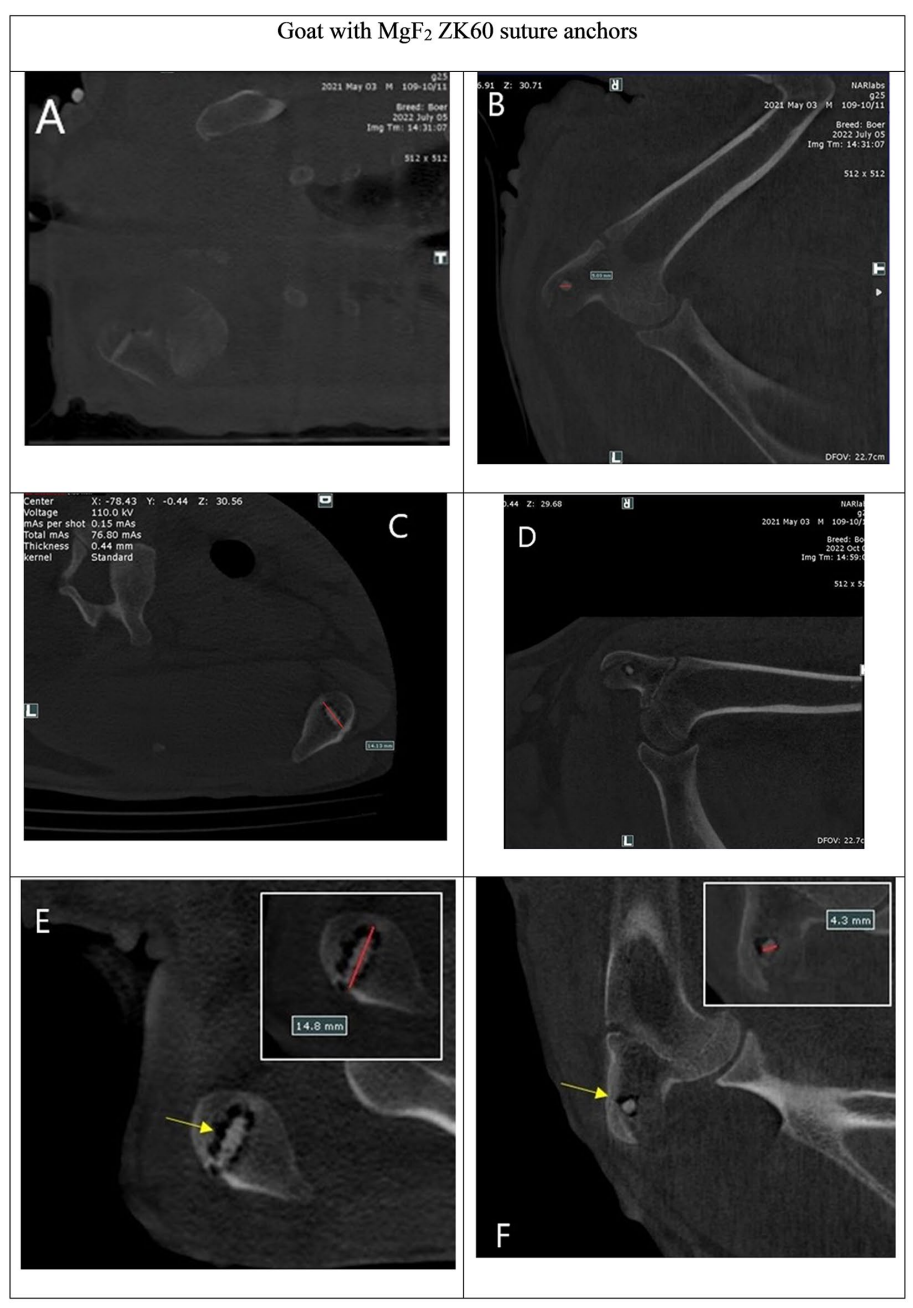
A series of consecutive CT images in goat implanted with ZK60 suture anchors. (**a**, **b**) Immediate postoperative, (**c**, **d**) 4-week postoperative, and (**e**, **f**) 12-week postoperative images. (**a, c, e**) sagittal plane, (**b, d, f**) coronal plane

**Fig. 6:**
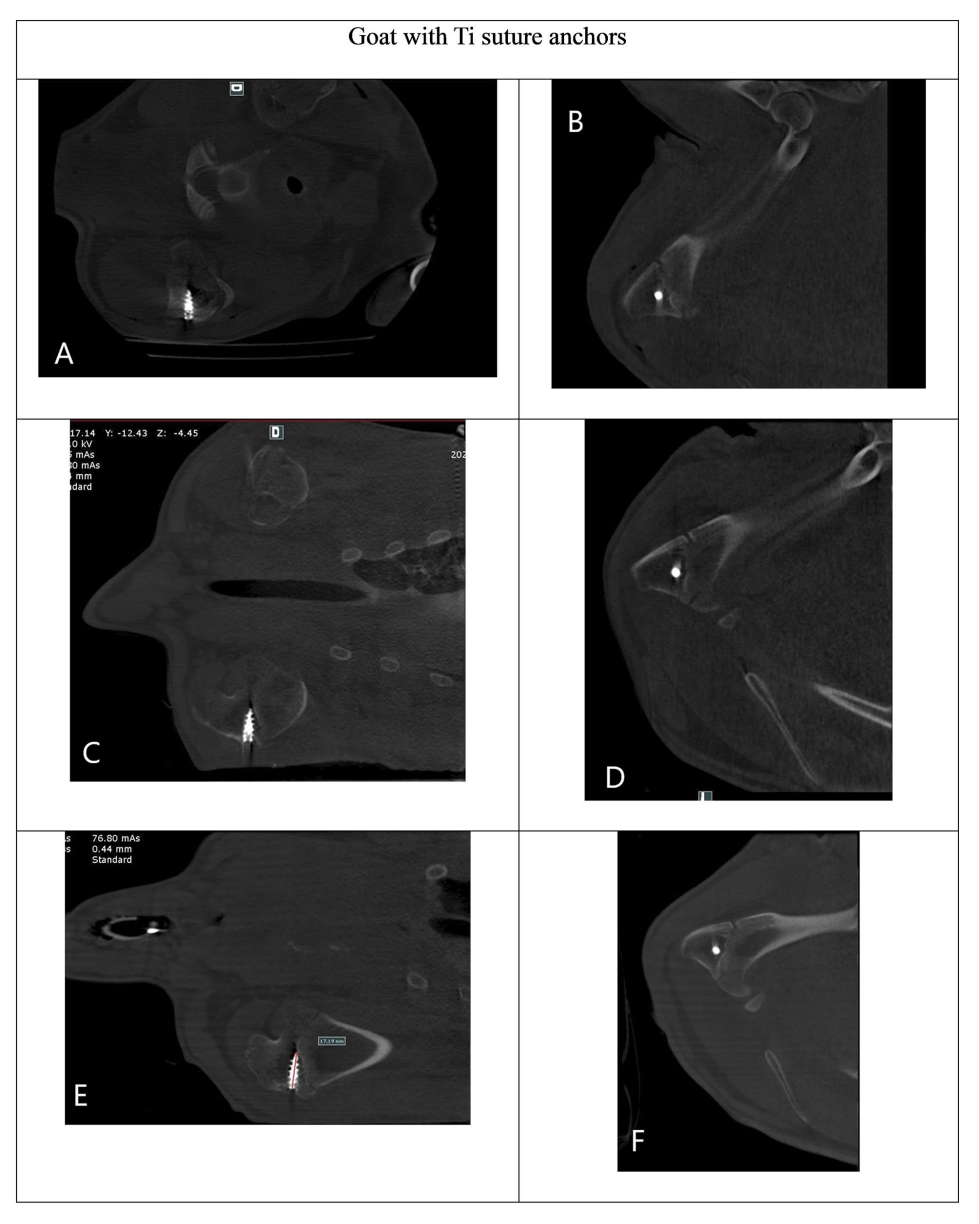
A series of consecutive CT images in goat implanted with Ti suture anchors. (**a**, **b**) Immediate postoperative, (**c**, **d**) 4-week postoperative, and (**e**, **f**) 12-week postoperative images. (**a, c, e**) sagittal plane, (**b, d, f**) coronal plane

The 12-week postoperative CT images revealed that the MgF_2_ ZK60 suture anchor maintained its structural integrity and effectively reestablished the connection between the detached infraspinatus tendon and the humeral head. Moreover, the anchors displayed a substantial decrease in visibility (Fig. 5) on CT scans, indicating their absorption by the surrounding tissues. Notably, the region of the MgF2 ZK60 anchor implantation was completely substituted with newly formed bone, which was denser than that in the Ti group (Fig. 6). Thus, the MgF_2_ ZK60 anchors demonstrated superior osseointegration to the Ti anchors. Moreover, the close similarity in mechanical properties between the cortical bone and magnesium may effectively mitigate the stress-shielding phenomenon while simultaneously providing adequate load-bearing capacity for the bone, promoting bone regeneration.

### Histological results

**Fig. 7:**
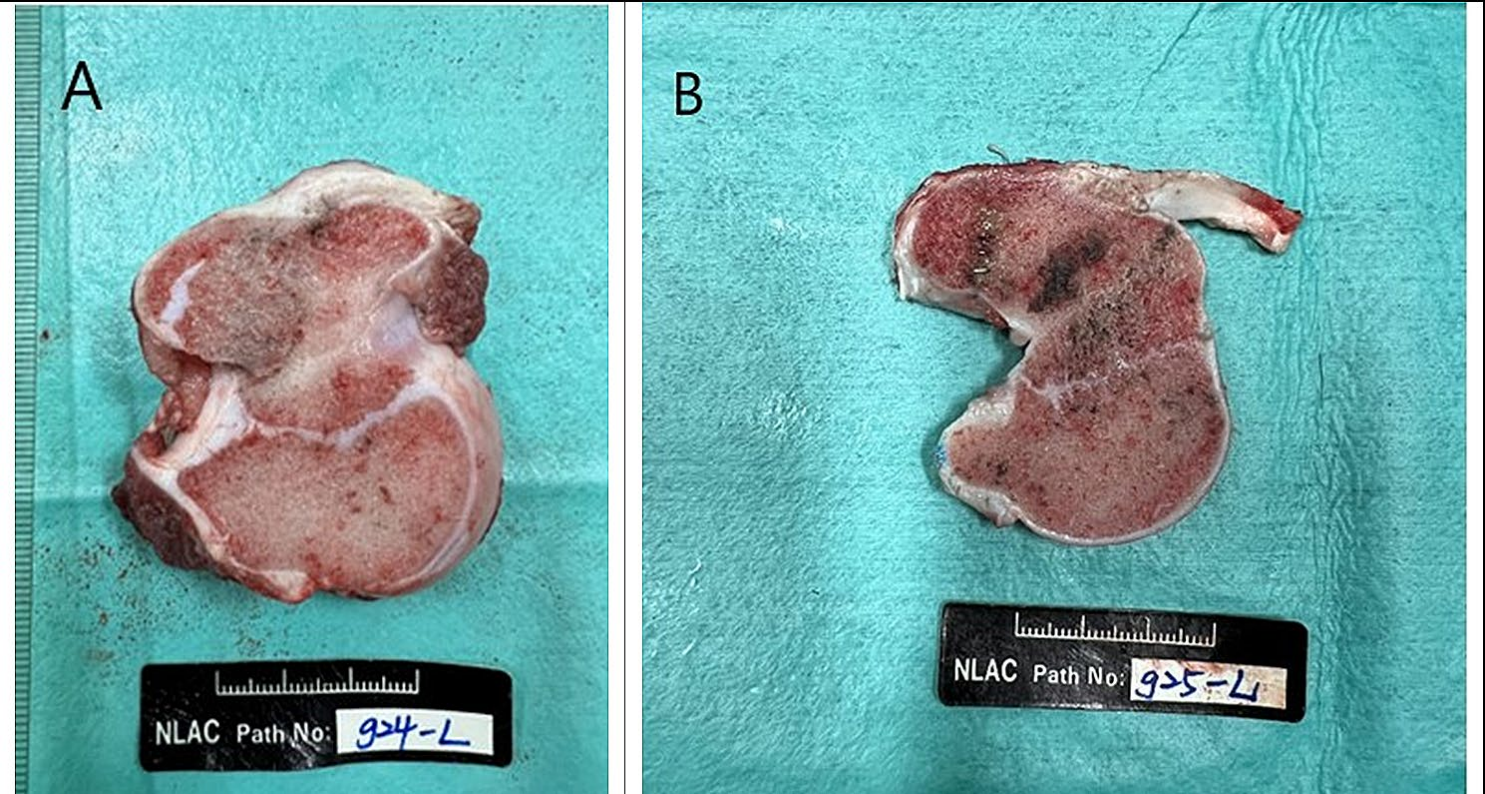
The comparison between goats implanted with ZK60 and Ti suture anchors. Proximal humerus specimen of (**a**) the goat that underwent Ti suture anchor implantation and (**b**) the goat that underwent MgF_2_ ZK60 suture anchor implantation. No gross abnormalities were observed in the bone specimens after the sacrifice at three months post-surgery for both the titanium alloy suture anchors and the magnesium alloy suture anchors

At 12 weeks postoperatively, the infraspinatus of the goats exhibited healing, and the bone–tendon junction appeared to be reestablished in both Ti and MgF_2_ groups. Macroscopic and histological evaluations were conducted to assess the bone–tendon repair zones and bone–implant interfaces at 12 weeks after the surgical repair procedure (Fig. 7). The tendon and bone samples and the implants were embedded for further analysis.

**Fig. 8:**
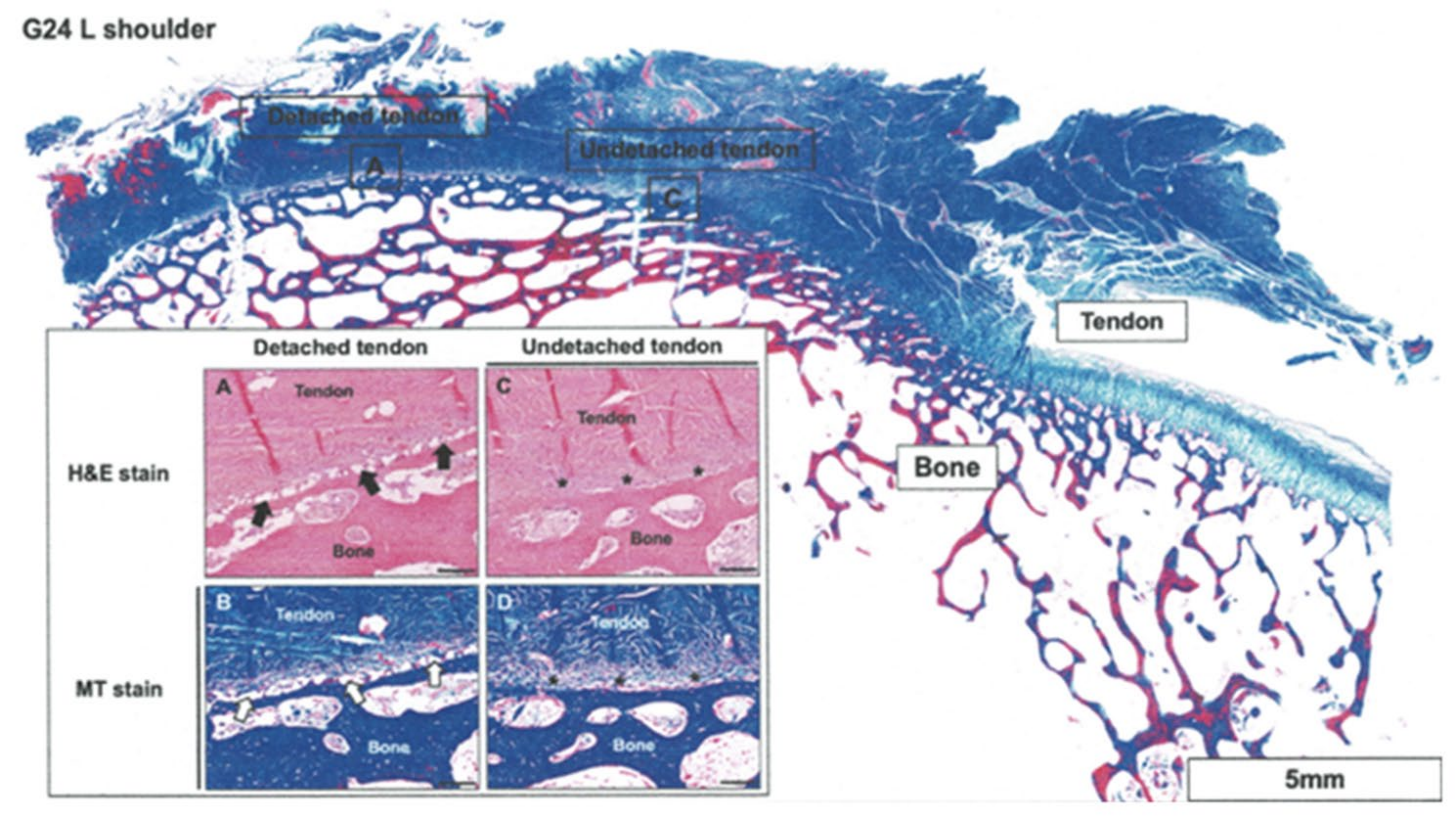
The histological analysis of goat implanted with Ti suture anchor. **a** Periosteum connecting the detached tendon and bone. Note the discontinuous interface with adipose infiltration (black arrows), which might reduce the tensile strength compared with the healthy tissue. H&E stain, scale bar = 100 μm. **b** Masson trichrome staining indicated collagen secretion on Fig. 1A, and the amount of collagen (white arrows) was reduced. MT stain, scale bar = 100 μm. **c** Periosteum of the undetached tendon revealed robust and continuous interface between the tendon and bone (*). H&E stain, scale bar = 100 μm. **d** Healthy tendons exhibiting dense and uniform collagen on the periosteum. MT stain, scale bar = 100 μm

Histological analysis of the Ti suture anchor at 12 weeks postoperatively revealed that the periosteum connecting the detached tendon and bone exhibited a discontinuous interface with adipose infiltration, which may result in reduced tensile strength compared with healthy tissue (Fig. 8). Masson trichrome (MT) staining revealed a decrease in collagen secretion, as indicated by the reduced amount of collagen. By contrast, the periosteum of the undetached tendon exhibited a robust and continuous interface between the tendon and bone. Healthy tendons displayed a periosteum with dense and uniformly distributed collagen.

**Fig. 9:**
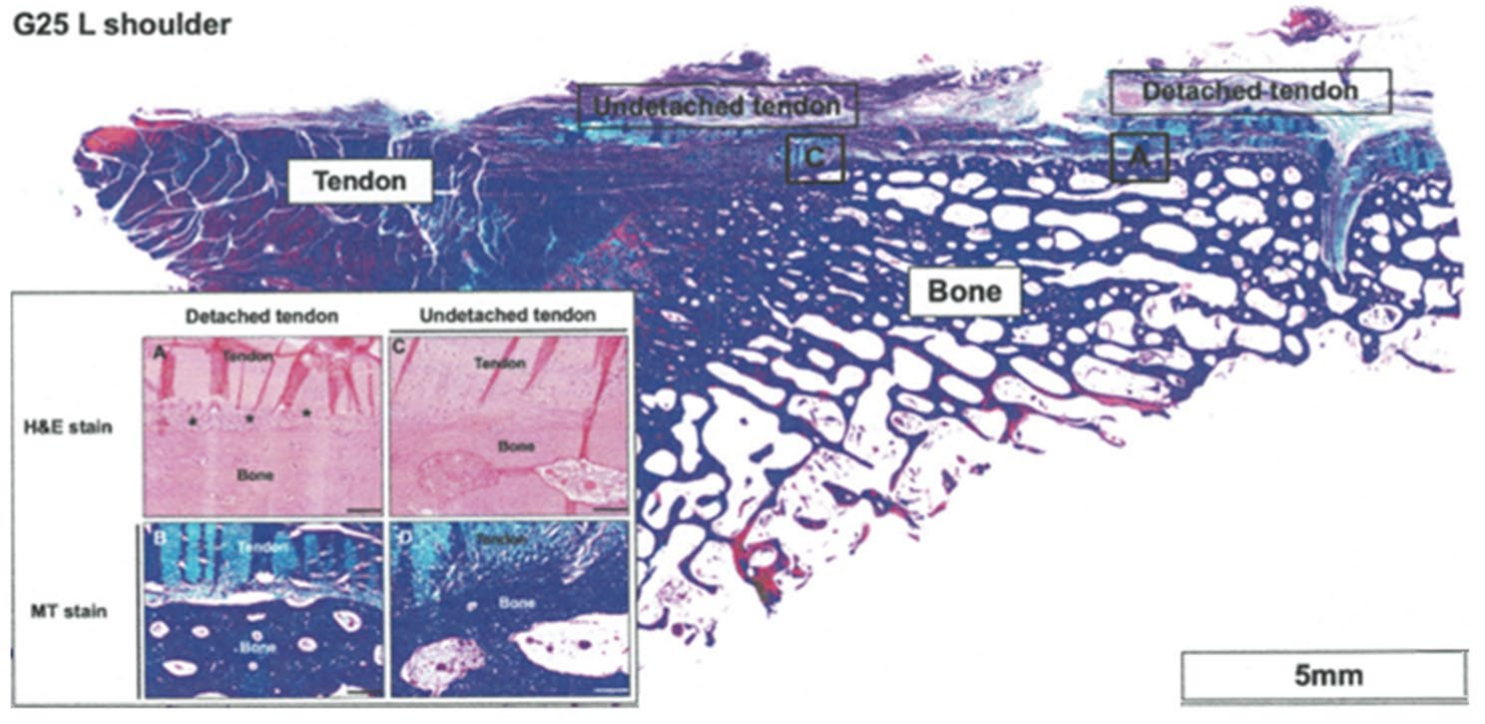
The histological analysis of goat implanted with MgF_2_ ZK60 suture anchor. **a** In detached tendon that repaired by MgF_2_ suture anchors, the periosteum was regenerated without any adipose infiltration as normal tissue (black arrows). H&E stain, scale bar = 100 μm. **b** These regenerated tendon and periosteum exhibited dense and uniform collagen on the periosteum. MT stain, scale bar = 100 μm. **c** Periosteum of the undetached tendon was intact without any fissure. H&E stain, scale bar = 100 μm. **d** Dense collagen on the tendon and periosteum. MT stain, scale bar = 100 μm

The periosteum of the detached tendon repaired using MgF_2_-coated suture anchors showed successful regeneration without adipose infiltration, resembling normal tissue (Fig. 9). The regenerated tendon and periosteum exhibited dense and uniform collagen distribution on the periosteum, indicating favorable healing. Similarly, the periosteum of undetached tendons remained intact without any fissures, suggesting the preservation of its structural integrity. Both the tendon and periosteum exhibited high collagen density, indicating a healthy and robust tissue composition. No significant differences were noted between the implant material scoring of the Ti and MgF_2_ ZK60 groups (Table 1).

**Table 1:** Histological evaluation format of cell and tissue response for implant material scoring

Group	Control			Test		
Implant material	Commercial Titanium suture anchors			Innovative MgF_2_ ZK60 suture anchors		
Observation period	Post-OP 3ms			Post-OP 3ms		
Animal code	G24			G25		
Area of interest	Infraspinatus tendon			Infraspinatus tendon		
Section code	L shoulder		R shoulder	L shoulder	R shoulder	
**H&E stain**						
Polymorphonuclear cells	1		0	1	0	
Lymphocytes	1		1	1	1	
Plasma cells	0		0	0	0	
Macrophages	1		0	1	0	
Giant cells	0		1	0	0	
Necrosis	0		0	1	0	
SUB-TOTAL (x2)	6		4	6	2	
Neovascularization	1		0	1	0	
Fatty infiltrate	2		0	0	0	
**MT stain**						
Fibrosis	3		4	4	4	
SUB-TOTAL	6		4	4	4	
GROUP TOTAL	12		8	10	6	
Average^a^	20/2 = 10.0			16/2 = 8.0		
		Test-Control = 0.0, no reaction				
No. Sites examined	2/2			2/2		

Post-OP 3 ms, 3 months postoperatively; H&E stain: hematoxylin and eosin; MT, Masson trichrome

^a^Used to determine reaction ranking shown below as he conclusion. A negative difference was recorded as zero

### Hematological examination

Hematological examination revealed that in the goat implanted with MgF_2_-coated ZK60 suture anchors, no abnormalities observed in liver and renal functions occurred at any point (Tables 2 and 3). The concentration of Mg ions remained stable (Table 4), and white blood cell, red blood cell, and platelet counts and coagulation function were within the normal range.

**Table 2:** Hematological examination of Goat with Ti suture anchors

Goat with Ti suture anchors					
	Unit	Preoperative	Postoperative	Post-OP 1 m	Post-OP 3ms
RBC	M/mcL	25.25	24	25.21	22
Hct	%	10.6	10.9	14	15.9
Hb	g/dL	10.4	10	10.7	10.3
MCV	fL	4.2	4.5	5.6	7.2
MCH	pg	4.1	4.2	4.2	4.7
MCHC	g/dL	98.1	91.7	76.4	64.8
% RETIC	%	0	0	0	0
RETIC	K/mcL	0	0	5	2.2
WBC	K/mcL	10.64	6.39	8.67	7.54
%NEU	%	47.7	34.4	43.7	39.6
%LYM	%	48.7	51.7	51.3	56.8
%MONO	%	2.6	3.4	3.1	1.7
%EOS	%	0	0.5	1.8	1.9
%BASO	%	0.1	0	0.1	0
NEU	K/mcL	5.07	2.2	3.78	2.99
LYN	K/mcL	5.18	3.94	4.45	4.28
MONO	K/mcL	0.28	0.22	0.27	0.13
EOS	K/mcL	0.1	0.03	0.16	0.14
BASO	K/mcL	0.01	0	0.01	0
PLT	K/mcL	614	610	592	532
MPV	fL	8.8	8.4	9.3	8

Post-OP 1 m, 1 month postoperatively; Post-OP 3 ms, 3 months postoperatively

RBC, red blood cell count; Hct, hematocrit; Hb, hemoglobin level; MCV, mean corpuscular volume; MCH, mean corpuscular hemoglobin; MCHC, mean corpuscular hemoglobin concentration; % RETIC, percentage of reticulocyte count; RETIC, reticulocyte count; WBC, white blood cell count; %NEU, percentage of neutrophils; %LYN, percentage of lymphocytes; %MONO, percentage of monocytes; %EOS, percentage of eosinophils; %BASO, percentage of basophils; NEU, neutrophil count; LYN, lymphocyte count; MONO, monocyte count; EOS, eosinophil count; BASO, basophil count; MPV, Mean platelet volume

**Table 3:** Hematological examination of Goat with MgF_2_ ZK60 suture anchors

Goat with MgF_2_ ZK60 suture anchors					
	Unit	Preoperative	Postoperative	Post-OP 1 m	Post-OP 3ms
RBC	M/mcL	28.97	28.6	25.99	25.7
Hct	%	6.3	7.7	4.8	8.6
Hb	g/dL	11.2	10.9	10	10.7
MCV	fL	2.2	2.7	1.8	3.3
MCH	pg	3.9	3.8	3.8	4.2
MCHC	g/dL	177.8	141.6	208.3	124.4
% RETIC	%	0	0	0	0
RETIC	K/mcL	0	0	0	0
WBC	K/mcL	9.64	13.48	10.3	9.84
%NEU	%	39.7	60	36.9	28.6
%LYM	%	55	35.3	55.5	63.1
%MONO	%	3.5	3.5	4	4.6
%EOS	%	1.8	0.1	3.5	3.7
%BASO	%	0	0.11	0.1	0
NEU	K/mcL	3.83	8.09	3.8	2.82
LYN	K/mcL	5.3	4.89	5.72	6.21
MONO	K/mcL	0.34	0.47	0.41	0.46
EOS	K/mcL	0.17	0.02	0.36	0.36
BASO	K/mcL	0	0.01	0.01	0
PLT	K/mcL	649	561	623	478
MPV	fL	8.8	8.7	8.8	8.3

Post-OP 1 m, 1 month postoperatively; Post-OP 3 ms, 3 months postoperatively

RBC, red blood cell count; Hct, hematocrit; Hb, hemoglobin level; MCV, mean corpuscular volume; MCH, mean corpuscular hemoglobin; MCHC, mean corpuscular hemoglobin concentration; % RETIC, percentage of reticulocyte count; RETIC, reticulocyte count; WBC, white blood cell count; %NEU, percentage of neutrophils; %LYN, percentage of lymphocytes; %MONO, percentage of monocytes; %EOS, percentage of eosinophils; %BASO, percentage of basophils; NEU, neutrophil count; LYN, lymphocyte count; MONO, monocyte count; EOS, eosinophil count; BASO, basophil count; MPV, Mean platelet volume

**Table 4:** Hematological examination of Goat with MgF_2_ ZK60 suture anchors

Goat with MgF_2_ ZK60 suture anchors					
	Unit	Preoperative	Postoperative	Post-OP 1 m	Post-OP 3ms
**Liver function**					
GOT	U/L	66	78	64	69
GPT	U/L	20	21	20	16
ALP	U/L	406	439	358	439
Bilirubin-total	mg/dL	0.21	0.43	0.1	0.06
γ-GT	U/L	61	55	54	57
**Renal function**					
BUN	mg/dL	28.9	32.4	21.3	16.2
Creatinine	mg/dL	1.39	1.52	1.16	0.98
Electrolyte					
Mg	mmol/dL	2.1	1.9	1.9	2.0

Post-OP 1 m, 1 month postoperatively; Post-OP 3 ms, 3 months postoperatively

GOT, glutamic oxaloacetic transaminase; GPT, glutamic pyruvic transaminase; ALP, alanine aminotransferase; γ-GT, gamma glutamyl transpeptidase; BUN, blood urea nitrogen

## Discussion

Based on clinical requirements, orthopedic implants can be broadly categorized into permanent and temporary implants. Permanent implants aim to replace severely damaged tissues or joints that are no longer functional, such as in joint replacement surgeries. Temporary implants are surgically implanted to provide a stable microenvironment for the self-repair of soft tissues, such as bones, tendons, and ligaments. Examples of temporary implants include plates used for bone fractures and the MgF2 ZK60 suture anchor used in our experiment. Their purpose is fulfilled once the soft tissues have successfully self-repaired. Ideally, these implants should disappear spontaneously to avoid subsequent clinical complications. For instance, titanium suture anchors used in rotator cuff repair surgeries cause metal artifacts in postoperative MRI, affecting image interpretation and hindering accurate medical decisions [1, 2]. To address the unmet need of temporary implants that spontaneously disappear, we designed and evaluated the use of degradable MgF_2_ ZK60 suture anchors for use in rotator cuff repair.

Various mechanical and biological factors influence the healing process of a ruptured rotator cuff tendon. Suture anchors can provide sufficient mechanical strength, provide a stable microenvironment, and promote rotator cuff tendon healing. However, concerns have been raised regarding the rapid degradation of magnesium alloys. If they degrade completely before tendon healing, they may fail to provide adequate stability, leading to further complications [4, 5]. Our previous data indicated that fluorination of the surface of the magnesium alloy implants effectively reduced the degradation rate when implanted in animals [14, 15]. Additionally, apart from the generation of hydrogen gas as a product of magnesium alloy degradation surrounding the implant, no abnormal immune reactions were observed in the tissues. This indicates the good biocompatibility of both the implant itself and its degradation products.

The results of the current study revealed that the release of magnesium ions during alloy degradation promoted new bone formation and enhanced bone integration compared with Ti suture anchors. This finding has significant implications for degradable magnesium alloy suture anchors. This is because if degradation occurs too rapidly, a large void may be formed in the bone where the anchors were implanted. Unless the surrounding tissue grows to fill this void, this can greatly impair the anchors’ mechanical strength and hinder tendon healing, thereby negating the advantages of the magnesium alloy anchors. A superior-quality implant should have the advantages of both titanium alloy anchors and degradable anchors—excellent mechanical strength with automatic disappearance without causing metal artifacts. In this regard, our results demonstrated that with the degradation of MgF2 ZK60 suture anchors, the released Mg ions promoted new bone formation, enabling the surrounding bone to grow and occupy the void, thereby ensuring that the mechanical strength is not compromised and facilitating tendon healing.

Hematological examinations revealed that the MgF_2_ ZK60 suture anchors did not adversely affect liver function, renal function, or hematopoiesis, indicating their safety. The variation in magnesium ion concentrations before, immediately after, and 1 and 3 months after surgery was minimal, indicating that the degradation proceeded at an expected rate. At 3 months postoperatively, these anchors remained in their original implanted positions and were not completely degraded. Thus, they do not seem to undergo excessively fast degradation.

Compared to Zhang et al. [13], our experimental subjects were large animals, instead of mice. We believe that the positive results obtained from large animal experiments would be more convincing to experts and scholars for further development of magnesium alloy suture anchors. Therefore, the most common surgical procedure in clinical practice involves using suture anchors to secure ruptured rotator cuff tendons to the greater tuberosity of the humerus. The fixation strength is sufficient to provide an optimal environment for rotator cuff tendon healing. However, it’s worth noting that Zhang. Et, al. used degradable magnesium alloy wire, which is rarely used in clinical settings due to concerns about insufficient fixation strength, potentially leading to poor healing outcomes. This aspect is supported by histological evidence showing the formation of lower-grade tendon components, namely fibrocartilaginous regeneration.

Chen et al [12]. uses goats as the subjects for animal experiments and employed histological, radiological, and hematological methods to assess the surgical outcomes of magnesium alloy suture anchors. These experimental designs align closely with our study. Additionally, it is possible that our magnesium alloy suture anchors underwent surface fluoridation treatment. Three months after the surgery, the magnesium alloy suture anchors in Chen et, al. appeared to have completely degraded, as they were barely visible on computed tomography scans, while our suture anchors still retained their magnesium alloy composition and were detectable in the scans.

In summary, the MgF_2_ ZK60 suture anchors used in this study have the following characteristics: (1) excellent biocompatibility, as evidenced by the absence of abnormal immune reactions in histology; (2) a relatively slow degradation rate, with magnesium ion concentrations remaining stable at 3 months postoperatively; (3) safety, as indicated by normal liver and kidney functions; and (4) sufficient mechanical strength, as evidenced by good tendon healing. Additionally, their degradation resolves the metal artifact issue in postoperative MRI scans.

On the basis of our results, we believe that the MgF_2_ ZK60 suture anchors have considerable potential for clinical applications and can overcome many of the challenges faced by clinicians. However, this study has some limitations. First, we used only two goats for this study, precluding quantitative analysis. Second, in clinical practice, rotator cuff tears can be small (< 1 cm), medium (1–3 cm), large (3–5 cm), or massive (> 5 cm). In this study, we created a 2-cm infraspinatus tear, which is a medium-sized tear, which required only a single suture anchor. Although the sizes of a goat’s humerus and infraspinatus muscle are similar to those of an adult human, clinically encountered tears in humans are often large, necessitating ≥ 2 anchors depending on the surgical technique used. Therefore, our experiment may have resembled the clinical scenario but did not fully replicate it. Further research, including human studies, are required to verify our results and provide additional supporting evidence.

As a preliminary study, our results indicate the potential utility of MgF_2_-coated anchors in orthopedic applications. Future studies are required to verify their clinical significance and their potential advantages in promoting rotator cuff tendon healing.

### Electronic supplementary material

Below is the link to the electronic supplementary material.

Supplementary Material 1

## Data Availability

The original data is available upon reasonable request to the corresponding author.

## Abbreviations

ZK60: Magnesium, zinc, and zirconium (Mg-6.0Zn-0.5Zr, ZK60) alloy
CT: Computed tomography
MgF2: Magnesium fluoride
Ti: Titanium
MRI: Magnetic resonance imaging
H&E staining: Hematoxylin–eosin staining
MT staining: Masson trichrome staining
Post-OP 3 ms: 3 months postoperatively
Post-OP 1 m: 1 month postoperatively
RBC: Red blood cell count
Hct: Hematocrit
Hb: Hemoglobin level
MCV: Mean corpuscular volume
MCH: Mean corpuscular hemoglobin
MCHC: Mean corpuscular hemoglobin concentration
% RETIC: Percentage of reticulocyte count
RETIC: Reticulocyte count
WBC: White blood cell count
%NEU: Percentage of neutrophils
%LYN: Percentage of lymphocytes
%MONO: Percentage of monocytes
%EOS: Percentage of eosinophils
%BASO: Percentage of basophils
NEU: Neutrophil count
LYN: Lymphocyte count
MONO: Monocyte count
EOS: Eosinophil count
BASO: Basophil count
MPV: Mean platelet volume
GOT: Glutamic oxaloacetic transaminase
GPT: Glutamic pyruvic transaminase
ALP: Alanine aminotransferase
γ-GT: Gamma glutamyl transpeptidase
BUN: Blood urea nitrogen
