# Supplementary material for: Fixation technique of biodegradable magnesium alloy suture anchor in the rotator cuff repair of the shoulder in a goat model: a technical note
Source: BMC Musculoskelet Disord. 2024 Mar 28;25:246. doi: 10.1186/s12891-024-07300-9 (PMC10976693; doi:10.1186/s12891-024-07300-9)
Supplement: Supplementary file 1 — Supplementary Material 1 [file 12891_2024_7300_MOESM1_ESM.docx]

**Supplementary Table 1** of ISO 10993-6:2016.

| **Cell type/response** | **Score** | | | | |
| --- | --- | --- | --- | --- | --- |
|  | **0** | **1** | **2** | **3** | **4** |
| Polymorphonuclear cells | 0 | Rare, 1 to 5/phf^a^ | 5 to 10/phf | Heavy infiltrate | Packed |
| Lymphocytes | 0 |  |  |  |  |
| Plasma cells | 0 |  |  |  |  |
| Macrophages | 0 |  |  |  |  |
| Giant cells | 0 | Rare, 1 to 5/phf | 3 to 5/phf |  | Sheets |
| Necrosis | 0 | Minimal | Mild | Moderate | Severe |

^a^ phf – per high-powered (400x) field

**Supplementary Table 2** of ISO 10993-6:2016

| **Response** | **Score** | | | | |
| --- | --- | --- | --- | --- | --- |
|  | **0** | **1** | **2** | **3** | **4** |
| Neovascularization | 0 | Minimal capillary proliferation, focal, 1 to 3 buds | Group of 4 to 7 capillaries with supporting fibroblastic structures | Broad band of capillaries with supporting fibroblastic structures | Extensive band of capillaries with supporting fibroblastic structures |
| Fibrosis | 0 | Narrow band | Moderately thick band | Thick band | Extensive band |
| Fatty infiltrate | 0 | Minimal amount of fat associated with fibrosis | Several layers of fat and fibrosis | Elongated and broad accumulation of fat cells about the implant site | Extensive fat completely surrounding the implant |
